# OA03.03. The effect of acupuncture on post cancer fatigue and wellbeing for women recovering from breast cancer: a feasibility randomized control trial

**DOI:** 10.1186/1472-6882-12-S1-O11

**Published:** 2012-06-12

**Authors:** C Smith, J Ussher, J Perz, B Carmady

**Affiliations:** 1 University of Western Sydney, Penrith South, Australia

## Purpose

Persistent and severe fatigue following breast cancer treatment is estimated to affect over a third of breast cancer patients. To date the evidence of effectiveness for the management of fatigue using acupuncture is unclear. We undertook a study to examine the feasibility and effect of acupuncture with managing fatigue in breast cancer survivors.

## Methods

We conducted a randomised controlled trial, comparing acupuncture with a placebo and a wait list control. Inclusion criteria included: aged between 18 and 70 years, a diagnosis of breast cancer, a score of >4 on the Brief Fatigue Inventory (BFI), and completed chemotherapy at least one month previously. Acupuncture was based on Traditional Chinese Medicine using a semi standardised treatment. The placebo control received non invasive needles using the Park Sham device, and placed away from points and meridians. Treatment was administered twice weekly over three weeks and weekly for three weeks. Endpoints were assessed at 2, 4 and 6 weeks, and included the BFI, Well-Being and Measure Yourself Concerns and Wellbeing questionnaire. In depth interviews were also conducted with women receiving acupuncture, exploring their sense of wellbeing, levels of fatigue and experience of acupuncture.

## Results

Thirty women were randomised and outcome data was available from 29 participants. At two weeks there was a significant reduction in fatigue for women receiving acupuncture compared to the placebo and wait list control (MD 5.3, 95% 4.5, 6.2, p=0.05). A trend to reduced fatigue for the acupuncture group was seen at week 4 (MD 4.6, 95% 3.6, 5.6, p=0.06), and at six weeks acupuncture demonstrated improved wellbeing (MD 2.7, 95% CI 2.2-3.2, p=0.006). Interviews demonstrated participants valued the therapeutic relationship, empowerment through self care and the invigorating nature of the intervention.

## Conclusion

The study demonstrated acceptability, feasibility and encouraging results to plan further research.

